# Bacterial communities within *Phengaris (Maculinea) alcon* caterpillars are shifted following transition from solitary living to social parasitism of *Myrmica* ant colonies

**DOI:** 10.1002/ece3.5010

**Published:** 2019-04-02

**Authors:** Mark A. Szenteczki, Camille Pitteloud, Luca P. Casacci, Lucie Kešnerová, Melissa R.L. Whitaker, Philipp Engel, Roger Vila, Nadir Alvarez

**Affiliations:** ^1^ Department of Ecology and Evolution University of Lausanne Lausanne Switzerland; ^2^ Museum and Institute of Zoology Polish Academy of Sciences Warsaw Poland; ^3^ Department of Life Sciences and Systems Biology University of Turin Turin Italy; ^4^ Department of Fundamental Microbiology University of Lausanne Switzerland; ^5^ Museum of Comparative Zoology Harvard University Cambridge Massachusetts; ^6^ Institut de Biologia Evolutiva (CSIC‐UPF) Barcelona Spain; ^7^ Unit of Research and Collections Museum of Natural History Geneva Switzerland; ^8^ Present address: Department of Environmental Systems Sciences Institute of Terrestrial Ecosystems, ETHZ Zürich Switzerland

**Keywords:** 16S amplicon sequencing, butterflies, Lepidoptera, Lycaenidae, myrmecophily, *Spiroplasma*

## Abstract

Bacterial symbionts are known to facilitate a wide range of physiological processes and ecological interactions for their hosts. In spite of this, caterpillars with highly diverse life histories appear to lack resident microbiota. Gut physiology, endogenous digestive enzymes, and limited social interactions may contribute to this pattern, but the consequences of shifts in social activity and diet on caterpillar microbiota are largely unknown. *Phengaris alcon* caterpillars undergo particularly dramatic social and dietary shifts when they parasitize *Myrmica* ant colonies, rapidly transitioning from solitary herbivory to ant tending (i.e., receiving protein‐rich regurgitations through trophallaxis). This unique life history provides a model for studying interactions between social living, diet, and caterpillar microbiota. Here, we characterized and compared bacterial communities within *P*. *alcon* caterpillars before and after their association with ants, using 16S rRNA amplicon sequencing and quantitative PCR. After being adopted by ants, bacterial communities within *P. alcon* caterpillars shifted substantially, with a significant increase in alpha diversity and greater consistency in bacterial community composition in terms of beta dissimilarity. We also characterized the bacterial communities within their host ants (*Myrmica schencki*), food plant (*Gentiana cruciata*), and soil from ant nest chambers. These data indicated that the aforementioned patterns were influenced by bacteria derived from caterpillars’ surrounding environments, rather than through transfers from ants. Thus, while bacterial communities are substantially reorganized over the life cycle of *P. alcon* caterpillars, it appears that they do not rely on transfers of bacteria from host ants to complete their development.

## 1 INTRODUCTION

Microbial symbionts can mediate diverse physiological processes in animals, particularly through adaptations that extend or enhance their trophic capacities. These symbioses can also lead to metabolic, developmental, and immunological adaptations in host animals, which facilitate their colonization of new environments and ultimately their evolution (McFall‐Ngai et al., 2013; Moran, 2002, 2007). Many insects also benefit from microbial symbioses, and their vast diversity in form and function may have arisen in part through associations with beneficial microorganisms, particularly bacteria (Engel & Moran, 2013). Recently, gut bacteria have been shown to enhance digestive capabilities (Brune, 2014; Kwong & Moran, 2016; Russell et al., 2009), protect against pathogens and predators (Koch & Schmid‐Hempel, 2011; Kwong, Mancenido, & Moran, 2017), and provide signals for inter‐ and intraspecific communication (Davis, Crippen, Hofstetter, & Tomberlin, 2013) and mating (Sharon et al., 2010) in insects.

Lepidopterans are a highly diverse order of insects, and their larvae (caterpillars) display diverse feeding habits ranging from general herbivory to obligate carnivory. Despite this dietary diversity, it appears that most lepidopterans typically host transient communities of bacteria derived from their food and surrounding environment (Berman, Laviad‐Shitrit, Lalzar, Halpern, & Inbar, 2018; Hammer, Mcmillan, & Fierer, 2014; Hernández‐Flores, Llanderal‐Cázares, Guzmán‐Franco, & Aranda‐Ocampo, 2015; Mason & Raffa, 2014; Phalnikar, Kunte, & Agashe, 2018; Robinson, Schloss, Ramos, Raffa, & Handelsman, 2010; Staudacher et al., 2016; Tang et al., 2012). Recently, Whitaker, Salzman, Sanders, Kaltenpoth, and Pierce (2016) found no clear link between trophic regime and gut bacterial composition, despite sampling a wide range of feeding strategies across 31 species of Lycaenid caterpillars. Hammer, Janzen, Hallwachs, Jaffe, and Fierer (2017) similarly found low densities of microbes in the guts of caterpillars spanning 124 species and 15 families.

Transient bacteria, which are excreted shortly after they are ingested with food, may dominate bacterial communities within caterpillars due to both physiological and ecological limitations. Highly alkaline conditions in the gut, coupled with relatively short and simple gut structures and a continuously replaced gut lining may limit or prevent the colonization of resident bacteria in caterpillars (Hammer et al., 2017). Development through several larval instars and metamorphosis may also dramatically reshape caterpillar digestive systems and any bacterial communities within them (Chen et al., 2016; Hammer et al., 2014). Moreover, many Lepidopterans engage in few social interactions outside of mating. This largely asocial development may also contribute to the apparent lack of beneficial resident bacteria within caterpillars, though until now, this has not been tested.

While social interactions may be uncommon for most caterpillars, many Lycaenid caterpillars engage in highly specialized interactions with eusocial ants. It is estimated that 75% of the approximately 6,000 Lycaenid species display some degree of myrmecophily (i.e., association with ants; reviewed in Pierce, 1995 and Pierce et al., 2002). These are usually facultative mutualistic interactions, in which ants protect caterpillars from predators and parasitoids in exchange for nutritive secretions. However, obligate parasitic associations also occur in a small subset (<5%) of myrmecophilous Lycaenid species (Pierce et al., 2002), including in the genus *Phengaris* (formerly *Maculinea*). Parasitic *Phengaris* caterpillars enter host ant colonies and feed either through ant regurgitations (trophallaxis), or by directly preying upon ant larvae.

Our focal species is the Alcon blue (*Phengaris alcon*), a widely studied parasitic Lycaenid species with a “cuckoo” feeding strategy. *P. alcon* caterpillars of the xeric ecotype (Koubínová et al., 2017) spend instars I–III (10–15 days) feeding on *Gentiana cruciata *buds. During the fourth instar, they fall off their host plant and are adopted by *Myrmica* worker ants, typically *Myrmica schencki* (Witek et al., 2008), though host ants can vary across the species distribution (Tartally, Nash, Lengyel, & Varga, 2008). Caterpillars utilize a combination of chemical (Akino, Knapp, Thomas, & Elmes, 1999; Nash, Als, Maile, Jones, & Boomsma, 2008) and acoustic (Barbero, Thomas, Bonelli, Balletto, & Schonrogge, 2009; Sala, Casacci, Balletto, Bonelli, & Barbero, 2014) signals to communicate with ants and avoid aggression, living in the colony for 1–2 years before pupating and emerging from the nest as an adult.

While living inside ant colonies, *P. alcon* caterpillars are dependent on regurgitations from host ant workers for nutrition. These regurgitations are rich in protein; *M. schencki *regularly consume other ants, as well as honeydew, nectar, and pollen (Czechowski, 2008). Regurgitations can be tailored to suit the nutritional needs of ant larvae (Dussutour & Simpson, 2009), and worker ants can play a role in the digestive processes of larvae directly, or by transferring beneficial gut symbionts with their regurgitations (Brown & Wernegreen, 2016). Consequently, when *P. alcon *rapidly shift from plant feeding to protein‐rich ant regurgitations, they may be able to enhance their survival and integration within ant colonies by exploiting bacterial transfers from their ant hosts.

Here, we leverage the asocial‐to‐social transition of *Phengaris alcon* caterpillars and the associated shift in diet to test whether obligate myrmecophily reshapes their bacterial communities. To address this question, we surveyed populations of wild *P. alcon* caterpillars, both while they were feeding on *G. cruciata *buds and after they had entered *M. schencki* colonies*,* using high‐throughput 16S rRNA amplicon sequencing. We also sequenced the bacterial communities within worker ants and ant larvae, and the surrounding environments of caterpillars (i.e., *G. cruciata* buds, and soil from inside ant nest chambers), to better understand the origins of any microbes present within caterpillars. Additionally, we used quantitative PCR to determine the total quantities of bacteria within *P*. *alcon* caterpillars and to test whether the number of bacteria within caterpillars shifted following their transition to living inside ant colonies. Together, these allowed us to fully assess the significance of bacterial symbioses as part of *P. alcon* caterpillars’ complex life history.

## 2 METHODS

### 2.1 Sample collection

Samples were collected across the Alps (Switzerland and Northern Italy) and Pyrenees (Spain) mountain ranges between 2015 and 2016. We collected III instar *Phengaris alcon* caterpillars by dissecting *G. cruciata* buds and IV instar caterpillars by excavating *M. schencki* nests. All caterpillars were starved for 3–4 hr until they evacuated their gut contents, and were then individually preserved in RNAlater^®^ (Thermo Fischer Scientific) tubes. *M*. *schencki* workers and larvae were collected from all ant colonies hosting *P. alcon* caterpillars, and were starved, preserved, and stored under the same conditions as caterpillars. Environmental samples (whole *G. cruciata* buds that caterpillars were eating, and 250 mg of fresh soil from ant nest chambers containing caterpillars) were collected in tandem with the above samples and frozen at −80°C until extraction.

### 2.2 16S rRNA amplicon processing

DNA extraction, library preparation, and preprocessing steps are detailed in Supporting Information Appendix S1. To summarize, we (a) extracted bacterial DNA from surface‐sterilized whole individuals, (b) amplified the V3/V4 region of the 16S rRNA gene in each sample, and (c) produced MiSeq‐compatible libraries for 300 bp paired‐end sequencing. Following these initial steps, we trimmed reads to 400 bp and performed open‐reference OTU picking in QIIME v.1.9.1 (Caporaso et al., 2010), using UCLUST (Edgar, 2010) to cluster OTUs at 97% identity. We filtered out probable chimeric sequences using UCHIME (Edgar, Haas, Clemente, Quince, & Knight, 2011) and the GOLD reference database (Reddy et al., 2015).

We assigned taxonomies using UCLUST and two reference databases: Greengenes v13_8 (DeSantis et al., 2006; McDonald, Price et al., 2012a) and SILVA NR Small Subunit v128 (Quast et al., 2013). We then used QIIME to filter out low abundance OTUs (i.e., with fewer than two reads) and over‐represented sequences (*Gentiana* chloroplast DNA and *Wolbachia*), produce biom (McDonald, Clemente et al., 2012b) tables for both the Greengenes‐ and SILVA‐annotated datasets, and create a phylogenetic tree using FastTree (Price, Dehal, & Arkin, 2009).

### 2.3 16S rRNA amplicon diversity analyses

QIIME outputs (biom tables, phylogenetic trees, and map files) were imported into R (R Core Team, 2017) for analysis using the *phyloseq* v.1.22.3 package (McMurdie & Holmes, 2013). First, we visualized bacterial community compositions among all groups of samples using bar plots. Then, we compared alpha (Shannon) diversities of *P. alcon* caterpillars on plants and inside ant colonies using a nonparametric two‐sample *t* test, with 1,000 Monte Carlo permutations. Next, we investigated whether the trophic shift and social association experienced by caterpillars in ant colonies led to more consistent bacterial communities, using assessments of beta dissimilarity. For these analyses, we rarefied the raw Greengenes‐annotated biom tables to even sampling depth (1,000 reads per sample), calculated Bray–Curtis and unweighted UniFrac distance matrices and visualized the results with nonmetric multidimensional scaling (NMDS) and principal coordinate analysis (PCoA) ordinations, respectively.

### 2.4 Determining the origins of bacterial communities within *P. alcon* caterpillars

Our final set of analyses using the 16S rRNA amplicon sequencing data investigated the relative contributions of social interactions and the environment on bacterial community composition and stability within *P. alcon* caterpillars. For these analyses, we CSS‐normalized (Paulson, Stine, Bravo, & Pop, 2013) the raw Greengenes‐annotated biom table using QIIME and used hclust2 (Segata, 2017) to visualize differences in abundances among the 40 most abundant OTUs (in terms of total read counts), clustering samples and features using Bray–Curtis dissimilarity. Then, we extracted the representative (i.e., most abundant) sequences for these 40 OTUs and performed BLAST searches of the NCBI nucleotide collection and 16S rRNA gene sequence databases to further improve the resolution of taxonomic identifications where possible. Then, we identified OTUs present in worker ants and caterpillars but not soil (i.e., OTUs that may have been exchanged between insects rather than environmentally derived) using the *shared_phylotypes* function in QIIME.

To determine which OTUs had the highest probability of being differentially abundant between all groups of caterpillars and ants, we performed a *G*‐test on the CSS‐normalized dataset using QIIME. To test for an effect of geography on the observed abundances, we repeated the *G*‐test using sample sites to group caterpillar and ant samples. We also used a Wilcoxon rank sum test to test for an effect of geography across caterpillars from Switzerland and Italy. All of the above‐mentioned tests included Bonferroni correction for multiple testing.

Finally, we searched for differentially abundant bacteria with possible digestive roles within *P. alcon* caterpillars using PICRUSt (Langille et al., 2013). For these analyses, we generated a closed‐reference OTU table from the Greengenes‐annotated, CSS‐normalized, dataset, and predicted metagenomic functions of OTUs in the form of KEGG Orthologs (Kanehisa & Goto, 2000). Then, we tested for the presence of differentially abundant features between caterpillars on plants and caterpillars in ant colonies using LEfSe (Segata et al., 2011).

### 2.5 Quantitative PCR analyses

We assessed whether the total quantities of bacteria within *P. alcon* caterpillars shifted following their association with ants using quantitative PCR. Using universal 16S rRNA primers, we determined the absolute and relative quantities of total bacteria within individual caterpillar and ant samples. Additionally, we determined the quantities of *Wolbachia* and *Spiroplasma *species present within caterpillars and ants using custom primers, based on the sequences present in our 16S rRNA amplicon sequencing dataset. All primers, PCR conditions and additional details on absolute and relative quantification methods are detailed in Supporting Information Appendix S1.

## 3 RESULTS

Among our three sampling locations (Supporting Information Appendix S2: Figure S1), we successfully sampled *P. alcon* caterpillars before and after their trophic shift at two sites (Switzerland and Italy). We were unsuccessful in locating caterpillars within ant colonies in Spain, but still sampled and sequenced caterpillars feeding on plants (*n* = 4) there. We sampled similar numbers of caterpillars on plants in Switzerland and Italy (*n* = 4 and *n* = 5, respectively). We found caterpillars within one ant colony in Switzerland (*n* = 4), and within two ant colonies at the same site in Italy (*n* = 2 and *n* = 3). Total numbers of samples for each group are detailed in Supporting Information Appendix S3, Table S1.

We identified 27,630 operational taxonomic units (OTUs) in the Greengenes‐annotated 16S rRNA amplicon sequencing dataset, and 28,504 OTUs in the SILVA‐annotated dataset. Excluding the environmental samples (*G. cruciata *buds and soil), there were 2,293 and 2,102 OTUs in the Greengenes and SILVA datasets, respectively. Initial exploratory analyses revealed that the Greengenes taxonomic identifications were generally of higher resolution than those produced using SILVA, with more genus‐level identifications and fewer unidentified OTUs. Thus, all results presented below will be based on Greengenes taxonomic identifications. However, we note that the SILVA‐annotated dataset produced similar results overall (Supporting Information Appendix S2: Figure S2).

### 3.1 The *P. alcon* trophic shift coincides with a shift in bacterial communities

**Figure 1:**
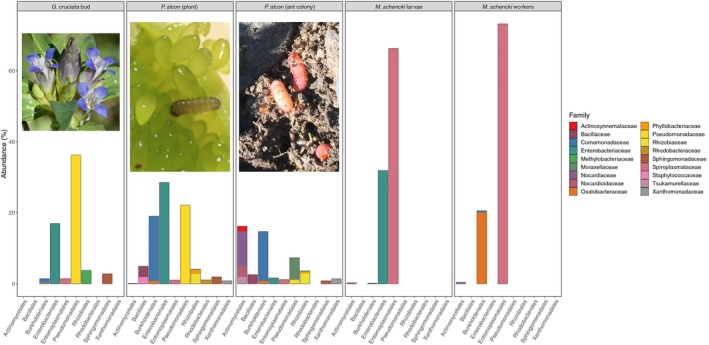
Bacterial community composition within *Phengaris alcon* caterpillars and *Myrmica schencki* workers and larvae. There is a clear shift in community composition following *P. alcon* caterpillars’ transition to parasitizing *M. schencki* colonies. We observed notable decreases in the abundances of Pseudomonadaceae and Enterobacteriaceae and an increase in Actinomycetales following caterpillars’ transition to living inside ant colonies. Note: average relative abundances for each group, across the top 40 OTUs in terms of total read count (62.9% of the total dataset) are shown above

Bar plot summaries of the 40 most abundant OTUs, which together represent 62.9% of all reads our final dataset, are shown in Figure 1. Bacterial communities within *P. alcon* caterpillars feeding on *G. cruciata *buds were dominated by Enterobacteriaceae (28%), Pseudomonadaceae (23%), and Comamonadaceae (18%). After caterpillars transitioned to living inside ant colonies, Enterobacteriaceae and Pseudomonadaceae decreased in abundance to 1.5% and 1.1%, respectively, while bacteria in the order Actinomycetales (17%), particularly family Nocardiaceae (12%), increased in abundance. *M. schencki *workers were dominated by *Spiroplasma* (74%) and Oxalobacteraceae (20%), while *M. schencki *larvae hosted primarily *Spiroplasma* (66%) and Enterobacteriaceae (32%).

The transition from *Gentiana* buds to ant colonies led to a large shift in overall community composition within caterpillars. In Switzerland, only 29 of 266 OTUs (10.9%) were shared between *P. alcon* caterpillars on plants and in ant colonies. Similarly, 33 out of 381 OTUs (8.7%) were shared between both stages of caterpillar development in Italy. Only 16 OTUs were shared among all caterpillars in Switzerland and Italy; taxonomic identifications for all of these shared OTUs can be found in Table 1. Higher proportions of OTUs were shared among individuals at the same site and life stage, but unique OTUs within individual caterpillars were more frequent within caterpillars in ant colonies. In Switzerland, 21% of OTUs (44/205) were shared among all *P. alcon* caterpillars on plants and 14% of OTUs (30/207) OTUs were shared among all caterpillars in ant colonies. In Italy, 40% of OTUs (63/159) were shared among caterpillars on plants and 23% of OTUs (99/432) were shared among caterpillars in ant colonies.

**Table 1:** OTUs present in caterpillars throughout both life stages (i.e., both before and after their trophic shift and association with ants)

*Phengaris alcon* on bud & *P. alcon* in ant colony (shared OTUs found in CH only)	*P. alcon* on bud & *P. alcon* in ant colony (shared OTUs found in CH and IT)	*P. alcon* on bud & *P. alcon* in ant colony (shared OTUs found IT only)
1025949_Mesorhizobium 1040713_Corynebacterium 1062748_Mycobacterium 928766_Chitinophagaceae 4394913_Sediminibacterium 168031_Erwinia 280799_Tepidimonas 590099_Sphingomonas 1091060_Sphingomonas_yabuuchiae 569952_Roseateles_depolymerans 544356_Polaromonas 136015_Delftia 136485_Methylobacterium_adhaesivum	963779_Agrobacterium 1093466_Agrobacterium 829523_Phyllobacteriaceae 816470_Bacillus 161287_Spiroplasma 698961_Spiroplasma 759061_Enterobacteriaceae 783638_Enterobacteriaceae 778478_Enterobacteriaceae 646549_Pseudomonas 967275_Stenotrophomonas 331752_Ralstonia 1108960_Sphingomonas 1104546_Rhizobiaceae 68621_Delftia 637901_Delftia	620684_Mesorhizobium 593555_Gluconobacter 1012112_Solirubrobacteraceae 622212_Spiroplasma 109263_Pseudomonas 836096_Pseudomonas 287032_Pseudomonas 279231_Pseudomonas 61192_Oxalobacteraceae 382348_Achromobacter 572643_Sinobacteraceae 1052559_Sphingomonadaceae 1091060_Sphingomonas 336364_Rhizobiaceae 210485_Comamonadaceae 323364_Delftia 525648_Rhizobiales

Note

The left‐ and rightmost columns contain the shared OTUs unique to Switzerland and Italy (respectively), while the center column contains the shared OTUs found in both countries. These OTUs represent the approximately 10% of bacterial taxa that persisted in *P. alcon* caterpillars following their trophic shift. Based on the Greengenes taxonomic identifications given above, most appear to be transient, environmentally derived bacteria.

### 3.2 *Phengaris alcon* caterpillars in ant colonies host more diverse and consistent bacterial communities

**Figure 2:**
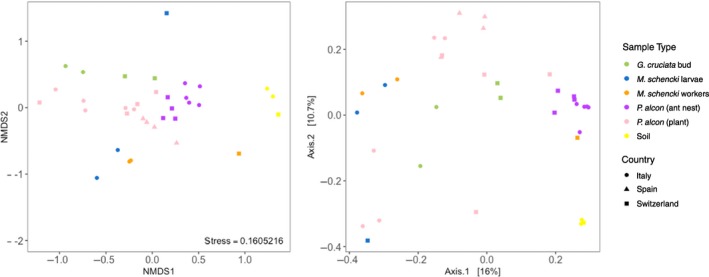
Multivariate representations of bacterial community composition (beta diversity), using nonmetric multidimensional scaling (NMDS) of Bray–Curtis dissimilarity (left) and principal coordinate analysis (PCoA) of unweighted UniFrac phylogenetic distances (right). *Phengaris alcon* caterpillars living in ant colonies (*n* = 9) appear to host more similar bacterial communities than caterpillars on plants (*n* = 13), in terms of beta dissimilarity. Note: both distance matrices were calculated from the Greengenes‐annotated dataset, with read counts rarefied to even sampling depth

We observed a significant increase in the alpha diversity of bacterial communities within *P. alcon *caterpillars living in ant colonies (Nonparametric two‐sample *t* test; *p* < 0.001). Caterpillars in ant colonies had the highest alpha (Shannon index) diversities, while caterpillars on plants appeared to be the most variable group (Supporting Information Appendix S2: Figure S3). In addition to producing more diverse bacterial communities within *P. alcon* caterpillars, the transition to living inside ant colonies also appeared to produce more consistent communities of bacteria in terms of beta diversity. In both Bray–Curtis/NMDS and unweighted UniFrac/PCoA ordinations, caterpillars on plants covered a wider area on the plots (i.e., were more dissimilar to one another) than caterpillars in ant colonies (Figure 2). This pattern was most pronounced when phylogenetic distances between OTUs were considered using UniFrac distances, though only 26.7% of the variance was explained by the first two axes of the PCoA. *M. schencki* workers and larvae also appeared to maintain distinct communities of bacteria, though ant samples from Switzerland did not cluster consistently.

### 3.3 *Phengaris alcon* caterpillars share many OTUs with their surrounding environments

**Figure 3:**
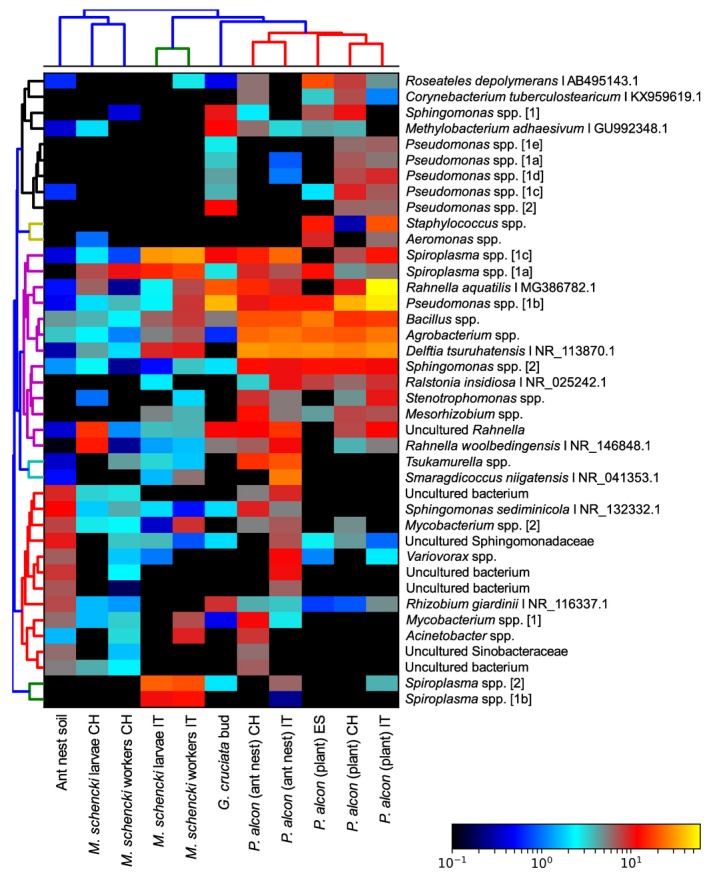
Heatmap of the 40 most abundant OTUs, with Bray–Curtis clustering of sample types (*X*‐axis; groups collapsed by averaging OTU abundances) and OTUs (*Y*‐axis). Environmental and/or pathogenic bacteria appear to account for most of the differentiation between *Phengaris alcon* caterpillars on plants and caterpillars in ant colonies. However, *Spiroplasma* species also appear to be useful in distinguishing between groups. Note: OTUs with >97% identity were denoted with subscripts (i.e., 1a/1b), while those with <97% identity were separately numbered.

While *P. alcon* caterpillars inside ant colonies appear to host more diverse and similar communities than caterpillars on plants, environmentally derived and putatively transient bacteria likely contributed to the above patterns; Swiss and Italian *P. alcon* caterpillars in ant colonies shared 79% and 87% of their total microbial diversity with ant nest soil, respectively. This result is also apparent when clustering groups based on the 40 most abundant OTUs in terms of total read counts (Figure 3). After manually confirming taxonomies of the most abundant bacteria using BLAST, we found that most of the highly abundant bacteria in our dataset are common on plants, or in soil and water (though we also note that bacteria with similar taxonomic identities can be adapted to different environments). When comparing bacterial abundances among all ants and caterpillars with a G‐Test, four OTUs (two *Spiroplasma*, a *Raoultella* species, and *Rahnella woolbedingensis*) were significantly differentially abundant (Supporting Information Appendix S3: Table S2) between groups. In contrast, no OTUs were significantly differentially abundant based on geographic location, in either the *G*‐test or the Wilcoxon rank sum test.

When considering OTUs shared among *P. alcon* caterpillars in ant colonies, *M. schencki* worker ants, and ant nest soil, almost all of the bacteria that were present within both ants and caterpillars (approx. 13% of all OTUs across these two groups) were also present in soil. In Switzerland, only five OTUs were found in caterpillars and ant workers, but not soil (*Bacillus* sp., *Delfita* sp., Nocardioidaceae, *Sphingomonas* sp., and *Spiroplasma* sp. 1). In Italy, eight OTUs shared between ant workers and caterpillars were not found in soil (*Achromobacter* sp., Actinomycetales, *Candidatus hamiltonella*, two species of *Delftia*, Isosphaeraceae, *Perlucidbaca* sp., and *Spiroplasma* sp. 2).

When comparing *P. alcon* caterpillars on plants to caterpillars in ant colonies, LEfSe analysis identified 52 significantly enriched KEGG orthologs among bacteria within caterpillars on plants and 48 significantly enriched KEGG orthologs among bacteria within caterpillars in ant colonies. However, few differentially enriched orthologs of caterpillars in ant colonies were parts of metabolic pathways (e.g., ko00071/Fatty acid degradation); the vast majority appeared to be unrelated to insect digestion (e.g., metabolism of several monoterpenoids, caprolactam, and naphthalene). Additionally, the most differentially enriched orthologs within caterpillars on plants appeared to be derived from free‐living, possibly pathogenic bacteria commonly found on plants (e.g., ko02030/Bacterial chemotaxis, ko03070/Bacterial secretion system, and dko00550/Peptidoglycan biosynthesis).

### 3.4 *Phengaris alcon* caterpillars host relatively small total quantities of bacteria

**Figure 4:**
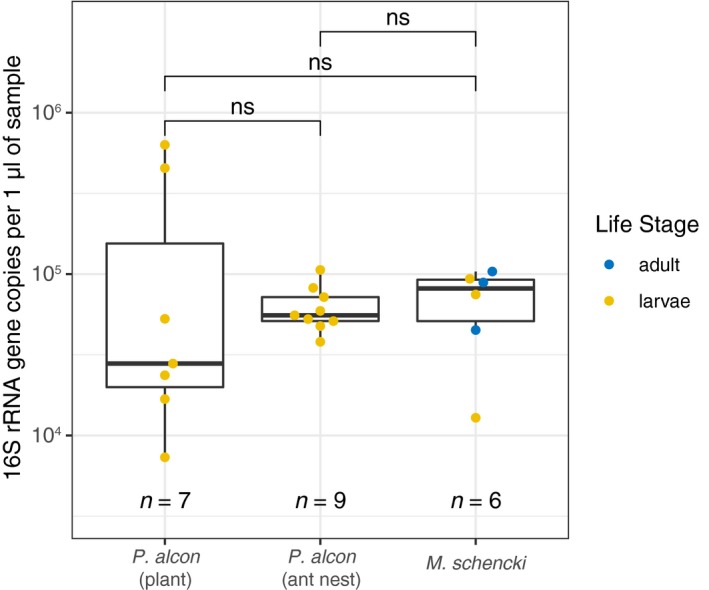
Boxplots representing total 16S rRNA gene copies per microlitre of DNA extraction in *Phengaris alcon* and *Myrmica schencki* samples. *P. alcon* caterpillars living inside ant colonies hosted more consistent, but not significantly different (Wilcoxon *p* > 0.05) total quantities of bacteria compared to *P. alcon* on plants. Note: two caterpillars living on plants from Switzerland, and all four caterpillars on plants from Spain are not shown above, as an insufficient quantity of DNA remained following 16S rRNA amplicon sequencing library preparation. Ant workers and larvae from the same nest were also (separately) pooled prior to extraction.

Consistent with Hammer et al. (2017), we also observed relatively low total quantities of bacterial DNA in all our caterpillar samples (Figure 4). We found an estimated 10^4^ bacteria per milligram of whole‐body tissue (Supporting Information Appendix S2: Figure S4), compared to ~10–10^4^ bacteria per milligram of gut tissue in larger caterpillar species (Hammer et al., 2017), placing *P. alcon* near the top of the range for quantities of bacteria known to be hosted by caterpillars. However, it should be noted that the total quantities of bacteria within *P. alcon* caterpillars, when scaled based on their size, are still lower than the quantities observed in other insects and animals.

Quantitative PCR analyses revealed that *P. alcon* caterpillars on plants hosted more variable, though overall not significantly different (Wilcoxon *p* > 0.05) absolute quantities of bacteria compared to caterpillars living in ant colonies (Figure 4). This variability within caterpillars on plants is also consistent with the patterns observed in our 16S amplicon sequencing data. When controlling for caterpillar size differences, we observed the same patterns in relative and absolute quantities of bacteria (Supporting Information Appendix S2: Figure S4). Using species‐specific qPCR primers, we also found that individual caterpillars and ants predominantly hosted either *Wolbachia *or *Spiroplasma* (Supporting Information Appendix S2: Figure S5).

## 4 DISCUSSION

Building on recent broad molecular surveys of microbial diversity within caterpillars (Hammer et al., 2017; Phalnikar et al., 2018; Whitaker et al., 2016), we characterized and compared bacterial communities within *Phengaris alcon* caterpillars before and after their trophic shift and social association with *M. schencki *ants. We observed a compositional shift (Figure 1), increase in diversity (Supporting Information Appendix S2: Figure S3), and homogenization (Figure 2) of bacterial communities within caterpillars following their transition to living inside *M. schencki* colonies. However, *M. schencki* workers and larvae shared relatively few bacteria with caterpillars living in their nests, and many of the most abundant bacteria within *P. alcon* were species common in soil and water (Figure 3). Taken together, these results imply that most bacteria within caterpillars are derived from their food and surrounding environment. These findings are consistent with other recent characterizations of Lepidopteran microbiota (Hammer et al., 2017; Phalnikar et al., 2018; Staudacher et al., 2016; Whitaker et al., 2016).

Quantitative PCR analyses were also generally consistent with the patterns observed in the 16S amplicon sequencing dataset. Notably, we did not detect significant differences in total quantities of bacteria when comparing between *P. alcon* caterpillars on plants with caterpillars in ant colonies (Figure 4). Our estimates of total bacterial abundances within caterpillars were near the upper bound reported in Hammer et al. (2017) (see Supporting Information Appendix S2: Figure S4). However, our *P. alcon* caterpillars weighed 50–100 times less than most caterpillars studied in Hammer et al. (2017); when accounting for this size difference, the total quantities of bacteria present within *P*. *alcon* caterpillars are still lower than in other similarly sized insects (see figure S3 in Hammer et al., 2017).

While Phalnikar et al. (2018) recently reported that bacterial communities within caterpillars (including two Lycaenidae) generally did not change during development, and that dietary transitions had weak effects on bacterial communities, our focused sampling (fully replicated across Switzerland and Italy) found a more substantial shift. Few “core” bacteria appear to persist over *P. alcon* caterpillar development; 8%–10% of OTUs persisted across both stages of development and both sampling sites (see Figure 1 and Table 1). However, none of the caterpillars in Phalnikar et al. (2018) underwent a trophic shift and change in environment as sudden and drastic as that experienced by *P. alcon* caterpillars. Furthermore, two Lycaenid species (*Leptotes plinius* and *Spalgis epius*) in Phalnikar et al. (2018) were not obligate myrmecophiles (Common & Waterhouse, 1972; Venkatesha, 2005). Given this result, we set out to disentangle the influence of diet, surroundings, and ant association on the diversity, structure, and origins of bacteria within *P. alcon* caterpillars.

In our initial comparisons of alpha diversities, we observed greater variability in bacterial community richness within *P. alcon* caterpillars on plants (Supporting Information Appendix S2: Figure S3). Some individuals were overwhelmingly dominated by one or a few bacteria not known to aid in digestion of plant material, suggesting that caterpillars do not crucially rely on metabolic associations with bacteria during most of their early development. This is not surprising, given that *P. alcon* caterpillars acquire 99% of their total biomass while living inside ant colonies (Thomas, Elmes, Wardlaw, & Woyciechowski, 1989). While some Lycaenidae are known to eat their eggshells, *P. alcon* caterpillars hatch basally, eating through the underside of the leaf their egg was laid on; they also do not eat their eggshells, which have an unusually thick protective chorion (Thomas, Munguira, Martin, & Elmes, 1991). This reduces the possibility for maternal transmission of bacteria to caterpillars, and thus it is likely that most bacteria within caterpillars on plants were derived from the *G. cruciata* buds they were eating.

While some *P. alcon* caterpillars on plants hosted diverse bacterial communities, many were dominated by Pseudomonadaceae, which include both plant‐growth promoting and pathogenic species (Preston, 2004) and Enterobacteriaceae, which include many common, harmless symbionts, but also pathogenic species. Enterobacteriaceae appear to be a common bacterial symbiont in Lycaenid larvae (Phalnikar et al., 2018; Whitaker et al., 2016).

In general, it would appear that the dominant groups of bacteria within *P. alcon* caterpillars in ant colonies are also derived from their surrounding environment, rather than through transfers from ants. Following the transition to living inside ant colonies, Pseudomonadaceae and Enterobacteriaceae decreased in abundance, while several families within the order Actinomycetales, particularly Nocardiaceae, increased in abundance (Figure 1). These bacteria are commonly found in soil and water (Goodfellow, 2014). The most abundant families in worker ants, Spiroplasmataceae and Oxalobacteraceae, were not similarly abundant within caterpillars. Caterpillars in ant colonies also hosted a greater diversity of bacteria than their host ants (see Figure 1 and Supporting Information Appendix S2: Figure S3). This implies a bacterial contribution from a source other than host ant regurgitations, such as soil. However, the lower diversity and quantities of bacteria within *M. schencki* may also be a consequence of more effective filtering of environmental bacteria, through immune defenses (Cremer, Armitage, & Schmid‐Hempel, 2007) or colonization resistance (Spees, Lopez, Kingsbury, Winter, & Bäumler, 2013).

Our measures of beta dissimilarity revealed that *P. alcon* caterpillars on plants could be highly dissimilar to one another, even within the same site (Figure 2). In contrast, caterpillars in ant colonies clustered more closely together and also clustered according to sampling location. Our qPCR data corroborated this finding, with caterpillars in ant colonies hosting more consistent (though not significantly greater) quantities of bacteria than caterpillars on plants (Figure 4). Taken together, these results suggest a homogenization of bacterial communities occurs within caterpillars following their transition to living inside ant colonies. Homogenous bacterial communities are a hallmark of highly social species (Shropshire & Bordenstein, 2016), and *P. alcon* caterpillars’ associations with ants seem to have led to consistent communities across a wide geographic range (i.e., across the Alps). However, environmentally derived bacteria likely remain the main driver of this pattern for *P. alcon* caterpillars (see Figure 3). This pattern may also be driven in part by relatively stable ant nest environments (Schär, Larsen, Meyling, & Nash, 2015) compared to plants, which can host diverse bacterial communities influenced by both biotic and abiotic factors (Bulgarelli, Schlaeppi, Spaepen, Themaat, & Schulze‐Lefert, 2013; Lindow & Brandl, 2003).

Given that *P. alcon* caterpillars in ant colonies shared 79%–87% of their OTUs with nest chamber soil, the observed shift in microbial communities following their transition from plants to ant colonies was certainly influenced by corresponding shifts in environmental bacteria. Some of these bacteria found in the environment could still have been acquired via trophallaxis, but we were unable to control for this when sampling wild populations of caterpillars. However, even with our more conservative analyses, further examination of the taxonomic identities of putatively transferred OTUs revealed that most were likely transient bacteria.

Some of the most consistently present bacteria in both caterpillars and ants are *Spiroplasma* and *Wolbachia*, two well‐known insect endosymbionts. Pathogenic strains of both *Spiroplasma* and *Wolbachia* are known to cause cytoplasmic incompatibility, feminization, and male killing. *Wolbachia* are very common parasites of lepidopterans (Salunkhe, Narkhede, & Shouche, 2014), and some *Spiroplasma* may play similar parasitic roles in lepidopterans (Jiggins, Hurst, Jiggins, v. d. Schulenburg, & Majerus, 2000). However, potentially mutualistic symbiotic effects have also been uncovered for both *Spiroplasma* (Jaenike, Unckless, Cockburn, Boelio, & Perlman, 2010; Xie, Vilchez, & Mateos, 2010) and *Wolbachia* (Bian, Xu, Lu, Xie, & Xi, 2010; Hedges, Brownlie, Oneill, & Johnson, 2008; Hosokawa, Koga, Kikuchi, Meng, & Fukatsu, 2010) in other insect groups. However, no such mutualisms between caterpillars and *Wolbachia* are currently known, so we considered *Wolbachia* to be an intracellular parasite only. Both *Wolbachia* and *Spiroplasma* can co‐occur within a host and have possible interactive effects on host immunity (Goto, Anbutsu, & Fukatsu, 2006; Shokal et al., 2016), though in our dataset, we observe a negative correlation between their abundances (Supporting Information Appendix S2: Figure S5). One explanation for this pattern is that *Spiroplasma* and *Wolbachia* may be respectively adapted to their ant and caterpillar hosts, and thus appear at lower abundances during cross‐infections.

*Spiroplasma* are known to be enriched among predatory ant species, including many *Myrmica* species (Anderson et al., 2012; Funaro et al., 2010). Recent research has also detected possible mutualistic *Spiroplasma* associations with *Myrmica*, which may aid in nutrient uptake and immunity (Ballinger, Moore, & Perlman, 2018). Transfers of these *Spiroplasma* from ants to caterpillars may therefore also aid in their digestion of regurgitated materials. Here, we detected two *Spiroplasma* with <97% identity (i.e., different strains/species), with some geographic variation in their abundances across Switzerland and Italy (see Figure 3). This may suggest local, long‐term mutualistic strains within host ants. However, our quantitative PCR results confirm that *Spiroplasma* are not highly abundant, and in some cases not present at all within caterpillars. Thus, transferred *Spiroplasma *are likely not essential to caterpillar digestion or survival. Furthermore, caterpillars on plants also contained small quantities of *Spiroplasma*, so several strains of *Spiroplasma* from both the environment and host ants may be present within caterpillars.

In addition to *Spiroplasma*, OTUs in the order Actinomycetales (e.g., Nocardioidaceae) were shared among ants and caterpillars in both Switzerland and Italy, but were not present in soil samples. Actinomycetales are known for their associations with leaf‐cutter ants, growing on specialized structures and protecting their hosts against parasites and pathogens (Barke et al., 2010; Currie, Poulsen, Mendenhall, Boomsma, & Billen, 2006; Haeder, Wirth, Herz, & Spiteller, 2009; Mattoso, Moreira, & Samuels, 2012). Actinomycetes with antifungal properties have also been identified in *Myrmica rugulosa* (Kost et al., 2007), and are a core component of the microbiota in other ants that do not farm fungi, such as *Pseudomyrmex* species (Rubin, Kautz, Wray, & Moreau, 2018). However, these bacteria are not currently known to enhance digestion in ants or caterpillars. Here, we found that that Actinomycetales are more abundant within caterpillars than ants (Figure 1); in fact, Actinomycetales account for <1% of all reads within ant workers and larvae. This may be due to our decision to surface sterilize both ants and caterpillars, which would eliminate bacteria colonizing the niche that Actinomycetales are most commonly associated with. However, surface‐sterilization also revealed that Actinomycetales colonize caterpillar gut (and other noncuticular) tissues more effectively than in ants. While it is possible that Actinomycetales may protect caterpillars and ants against pathogens in the ant nest environment, this difference in localization and abundance reduces the likelihood that they play identical roles in both caterpillars and ants.

## 5 CONCLUSION

Microbes are increasingly being recognized as having a strong influence on the evolution of sociality (Archie & Theis, 2011; Archie & Tung, 2015; Lombardo, 2008). However, it remains difficult to disentangle the influences of shared diets, shared environments, and social interactions on microbial communities without controlled, long‐term studies (e.g., Tung et al., 2015). We observed a homogenization of bacterial communities within *P. alcon* caterpillars following their social association with ants, and could identify possible transfers of a few species, notably *Spiroplasma *and Nocardiaceae, between ants and caterpillars. However, as observed in other caterpillars, the majority of bacteria characterized were not present in host ants, but were rather abundant in caterpillars’ food and surroundings (i.e., *G. cruciata* buds and ant nest chamber soil).

Ultimately, it appears that bacterial symbionts are not essential to *Phengaris alcon* caterpillars as part of their suite of adaptations for interacting with and parasitizing host ant colonies. However, antibiotic treatment experiments are needed to confirm whether adoption and survival rates within host ant colonies are influenced by bacterial communities. Endogenous genes and pathways within *P. alcon* caterpillars are likely essential for their interactions with ants. As Whitaker et al. (2016) recently suggested, some of the genes facilitating interactions between caterpillars and ants may also have been horizontally transferred from previous bacterial associations, but the genomes of *P. alcon *or their host ants have not yet been characterized. Given the data currently available, we favor a scenario in which the complex life history of *P. alcon* caterpillars can persist without any sustained symbiosis with microbes.

## CONFLICT OF INTEREST

None declared.

## AUTHOR CONTRIBUTIONS

MAS, CP, MRLW, RV, NA designed research; MAS, CP, LPC, RV performed research; CP, LK contributed new reagents or analytical tools; MAS, LK analyzed data; MAS, LPC, MRLW, LK, RV, NA wrote the paper.

## Data Availability

Raw 16S amplicon sequences, metadata, preprocessed BIOM tables, and qPCR data are available at http://doi.org/10.5061/dryad.60008mj.
