# Supplementary material for: Bacterial communities within Phengaris (Maculinea) alcon caterpillars are shifted following transition from solitary living to social parasitism of Myrmica ant colonies
Source: Ecol Evol. 2019 Apr 2;9(8):4452–64. doi: 10.1002/ece3.5010 (PMC6476763; doi:10.1002/ece3.5010)
Supplement: Supplementary file 1 [file ECE3-9-4452-s001.docx]

**APPENDIX S1 – SUPPLEMENTARY METHODS**

1. DNA extraction

*P. alcon* caterpillars and *M. schencki* workers/larvae were stored in RNAlater® (Thermo Fischer Scientific) at 4°C until extraction. All samples were extracted within two weeks of collection, using sterile techniques (i.e. under a laminar flow cabinet, with UV- and flame-sterilized equipment, and sterile consumables). RNAlater and debris were rinsed off samples with sterile water before extraction. Samples were then individually extracted whole (except for soil, were 0.25g thawed wet soil was used), using a MO BIO PowerSoil DNA Isolation Kit (Qiagen). The PowerSoil ‘experienced user protocol’ was followed, with the addition of 100μg of Proteinase K alongside solution C1, followed by an overnight digestion at 56°C and with 650rpm shaking. All caterpillar and environmental samples were extracted individually; however, 4-5 *M. schencki* individuals were pooled separately (i.e. for each nest, and for both workers and larvae) during DNA extraction to increase yields. Extraction concentrations were quantified using Qubit HS (Thermo Fischer Scientific) fluorimetry, and all samples were stored at -20 C until library preparation.

2. 16S rRNA amplicon library preparation and sequencing

All extractions were diluted to an equimolar ratio (5 ng/μl) using sterile filtered water (Sigma Aldrich) prior to library preparation. Then, we amplified the V3 and V4 regions (~460bp) of the bacterial 16S rRNA gene in the individual, diluted samples. We used custom Illumina-compatible versions of the universal 341F (5' – ACACTCTTTCCCTACAC GACGCTCTTCCGATCTCCTACGGGNGGCWGCAG - 3') and 805R (5' – GTGACTGGA GTTCAGACGTGTGCTCTTCCGATCTGACTACHVGGGTATCTAATCC - 3') primers for our amplicon PCR, which was carried out with the following conditions: 3 minutes of hot-start at 95°C, followed by 30 seconds at 95°C/55°C/72°C (each) for 25 cycles, and a 5 minute final extension at 72°C. Next, PCR products were purified with Agencourt AMPure XP (Beckman Coulter) at a 0.8X ratio, and individually indexed using standard Illumina indices, bringing the final length to ~580bp. The indexing PCR products were then purified again using AMPure at a 0.8X ratio. After determining the final concentrations of all samples using Qubit fluorimetry, samples were pooled into two sequencing libraries at equimolar ratios. Both libraries were sequenced at the Lausanne Genomic Technologies Facility (Lausanne, Switzerland) on an Illumina MiSeq, using 300bp paired-end sequencing.

3. Preprocessing of 16S rRNA amplicon sequencing data

The resulting forward and reverse reads had sufficient quality (min. Phred64 scores >30) and overlaps (>5bp) to be merged with USEARCH v7.0.1090 (Edgar 2010). We then trimmed the 16S primer sequences from the merged reads using USEARCH. Next, we removed any remaining reads that contained adapter and/or primer sequences using BBDuk (Bushnell 2017). We then used BBDuk to filter out two groups of extremely over-represented sequences not relevant to downstream analyses (*Gentiana* chloroplast DNA and *Wolbachia spp*.).

4. Quantitative PCR

All qPCR reactions were carried out in a 96-well plate on a QuantStudio5 instrument (Applied Biosystems) with the thermal cycling conditions as follows: denaturation stage at 50°C for 2 min followed by 95°C for 2 min, 40 amplification cycles at 95°C for 15 s, and 60°C for 1 min. Melting curves were generated after each run (95°C for 15 s, 60°C for 20 s and increments of 0.3°C until reaching 95°C for 15 s) to compare dissociation characteristics of the PCR products obtained from samples and positive controls. Each reaction was performed in triplicate in a total volume of 10 μl (0.2 μM of each forward and reverse primer or 0.4 μM of each universal bacterial primer due to the presence of ambiguous bases in the primer sequence; and 1x SYBR® Select Master Mix, Applied Biosystems) with 1 μl of DNA sample of known concentration (diluted to 10 ng/μl). Each plate contained a negative (water) control.

The MIQE guidelines (minimum information for publication of qPCR experiments) were followed throughout the data analysis of the qPCR experiments (Bustin et al. 2009). A uniform detection value of fluorescence intensity was set for each target and kept the same across all qPCR plates of the study. Technical outliers/errors from each triplicate were eliminated and mean quantification cycle (Cq, previously known as the threshold cycle, Ct; Bustin et al. 2009) and SD values were calculated. Then, the data was exported from the QuantStudio5 qPCR instrument for further processing in R.

To determine the absolute quantity of bacterial 16S rRNA genes in the samples we performed standard curves on serial dilutions of plasmids (pGEM®-T Easy vector; Promega) containing the 16S rRNA sequence of the honey bee gut associate *Gilliamella apicola* (used for universal bacterial primers) and *Wolbachia* and *Spiroplasma* from this study. The final concentrations of the plasmid in these template samples ranged from 10^7^– 10^1^copies per μl. The plasmid copy number was calculated from the molecular weight of the plasmid and the DNA concentration of the purified plasmid measured with a Qubit (Thermo Fisher) fluorometer. The slope and intercept of standard curves were calculated based on the Cq values obtained from the 10^7^–10^1^ copy dilutions. The E values were estimated from the slopes according to the equation: E = 10^(‑1/slope) (Pfaffl 2001). No amplification was obtained at the highest dilution (10 copies) in case of the universal bacterial primers. The Cq value at which amplification was detected for the lowest plasmid copy number was considered as the limit of detection (LOD) of a given primer pair. Primer characteristics and their performance are summarized in Appendix S3, Table S3.

We determined the number of bacterial 16S rRNA gene copies per 1 µl of DNA sample (Figure 4) for each DNA sample. The copy number of the bacterial target in 1 μl was calculated from the Cq value and the standard curve using the formula n = E^(intercept - Cq) (Gallup 2011). We also calculated the number of bacterial 16S rRNA gene copies per 1 mg of whole body tissue (Appendix S2, Figure S4). As caterpillars were not weighed prior to extraction, we used an average fresh weight of 10mg for caterpillars on plants and 20mg for caterpillars living inside ant colonies (Elmes et al. 1991). Then, we multiplied the number of bacterial 16S rRNA copies per 1 µl of sample by its total volume and subsequently divided by the sample weight (10 or 20 mg). While less precise, this approach allows us to compare other published bacterial loads per unit weight.

To confirm our results, we also used a relative comparison of bacterial loads in caterpillars from plant buds and ant colonies (Appendix S2, Figure S4). For this, we used amplification values of *P*.*alcon* ALIT3 elongation factor-1 alpha (EF1a) as an internal normalizer for host tissue quantity. The relative ratio considering differences in primer efficiencies was calculated based on the formula: Ratio = ((E_target_)^ΔCq, target (calibrator – test)^) / ((E_ref_)^ΔCq, ref (calibrator – test)^) [1]( Pfaffl, 2001), where the ‘target’ refers to values obtained when universal bacterial 16S primers were used and ‘ref’ when *P. alcon* EF1 gene primers were used. Median Cq value obtained for caterpillars on plant buds (calculated separately for the ‘target’ and the ‘ref’) were used as the ‘calibrator’ value. These indicated a relative increase of bacterial loads in the caterpillars parasitizing ant colonies, compared to the median of caterpillars eating plant material. However, the spread of values for plant-caterpillars resulted in non-significant difference when tested with Wilcoxon rank sum test.
