# Supplementary material for: Bacterial communities within Phengaris (Maculinea) alcon caterpillars are shifted following transition from solitary living to social parasitism of Myrmica ant colonies
Source: Ecol Evol. 2019 Apr 2;9(8):4452–64. doi: 10.1002/ece3.5010 (PMC6476763; doi:10.1002/ece3.5010)
Supplement: Supplementary file 2 [file ECE3-9-4452-s002.docx]

# APPENDIX S2 – SUPPLEMENTARY FIGURES


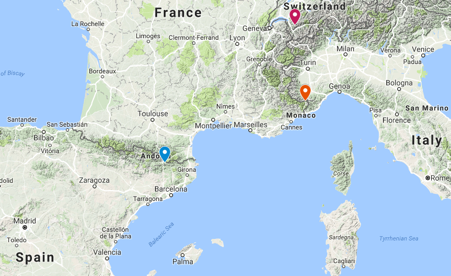


**Figure S1**| *Phengaris* (*Maculinea*) *alcon* sampling sites in Spain, Switzerland, and Italy.


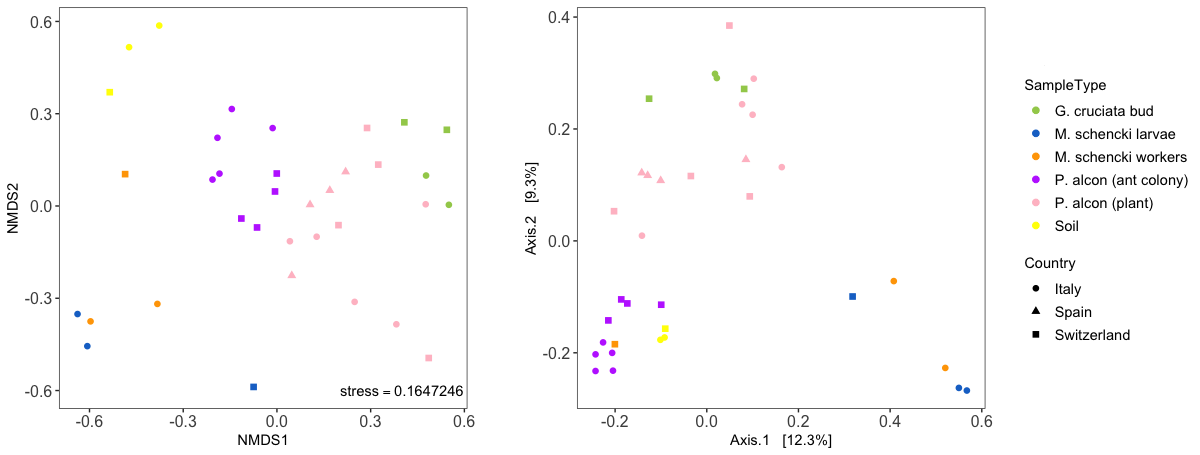


**Figure S2** **| Caterpillars in ant colonies also cluster more closely together than caterpillars on plants** when taxonomic identification was carried out using SILVA v128. Overall weaker differentiation between groups (but similar stress) was observed in the NMDS plots of Bray-Curtis dissimilarity (left). In the PCoA plot of unweighted UniFrac distances (right), the axes described a smaller portion of the variation, and soil clustered more closely with caterpillars living in ant nests. All samples were processed identically as the Greengenes-annotated data, and the raw data were rarefied to 1000 reads/sample prior to calculating distances between samples.


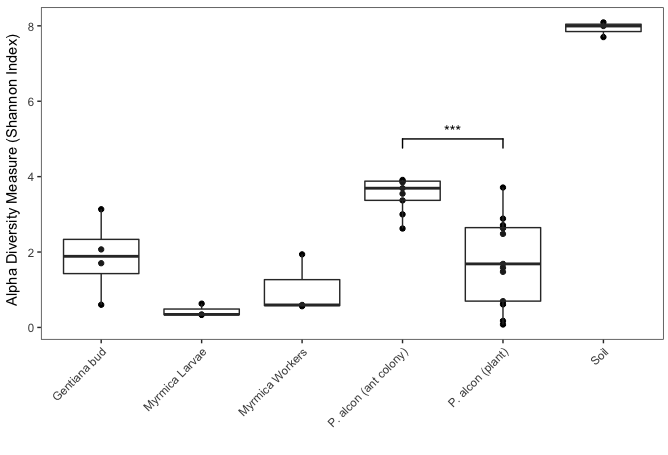


**Figure S3** | The alpha diversity (Shannon index) of bacterial communities within *P. alcon* caterpillars significantly increased (Nonparametric two sample t-test; p < 0.001), following their transition from living on plants to living inside *M. schencki* colonies. Ants also appeared to host less diverse bacterial communities than caterpillars in terms of alpha diversity.

**
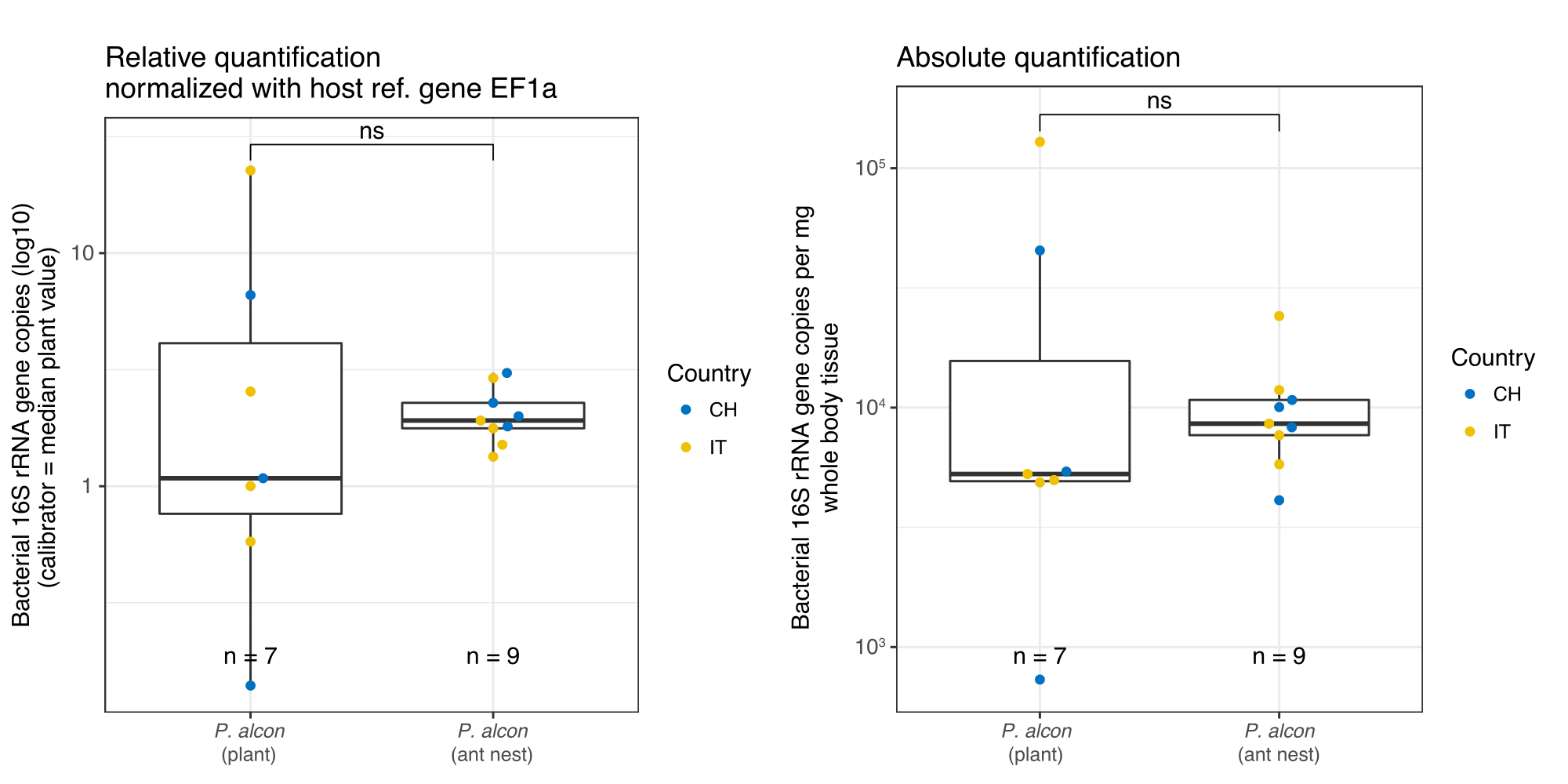
Figure S4** | After normalizing for body size using qPCR primers for *P. alcon* housekeeping gene EF1a and performing relative quantifications of bacterial loads (left panel), **caterpillars living in ant colonies still had more consistent, but not significantly different quantities of bacteria (Wilcoxon *p* > 0.05) than caterpillars on plants**. When normalizing absolute quantifications per mg of total body tissue using estimated weights for individuals (right panel), similar patterns were obtained. Note: wet weights had to be estimated from literature values (Elmes et al. 1991), as caterpillars were not weighed prior to extraction.

**
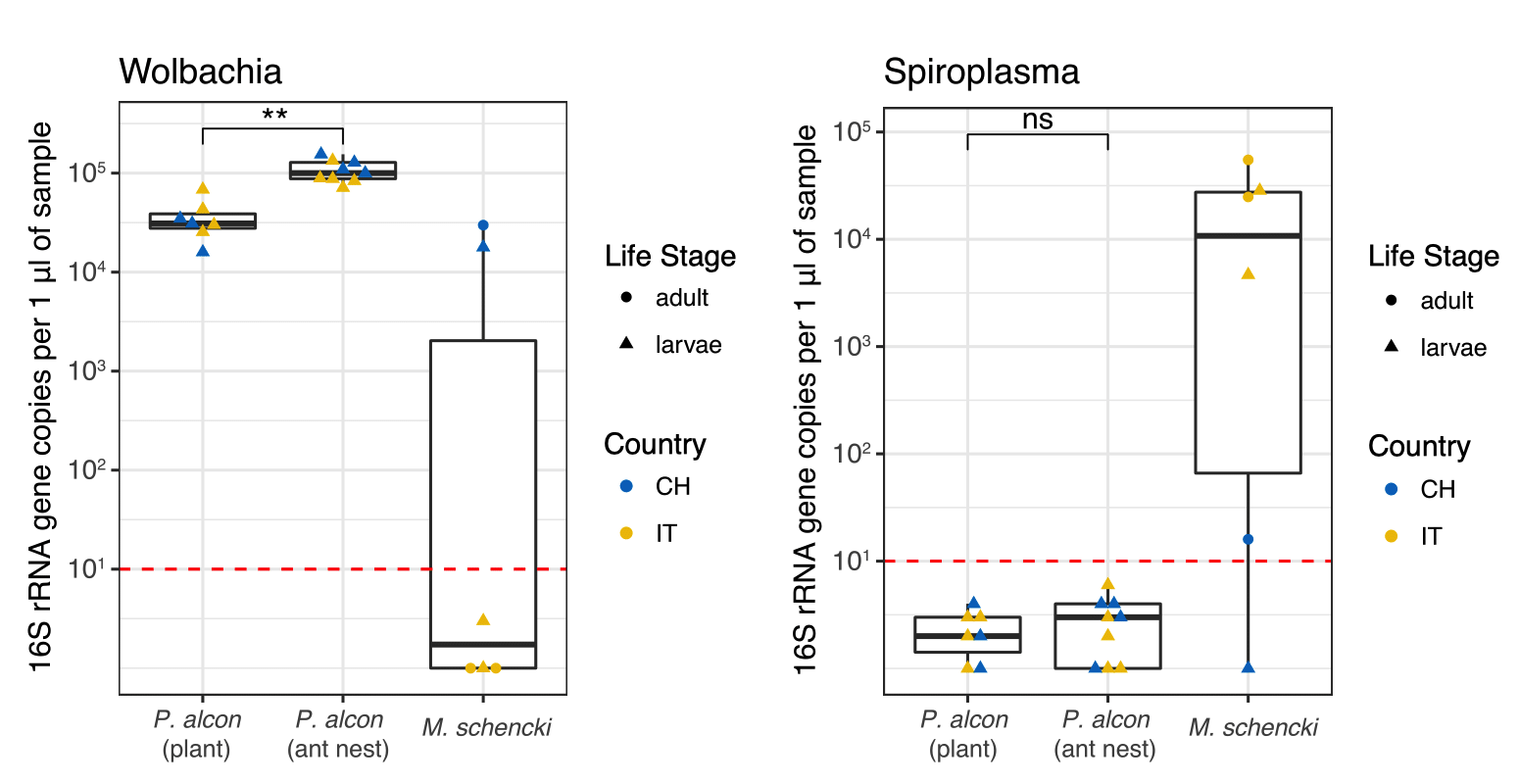
Figure S5** | 16S rRNA gene copy numbers for *Wolbachia-* and *Spiroplasma-*specific quantitative PCR reactions; *Wolbachia* are present in all caterpillars, and there is a significant increase in their abundance after caterpillars’ transition to living inside ant colonies (Wilcoxon *p* < 0.00103). *Spiroplasma* generally occurred at low abundance within caterpillars and were likely absent within some individuals. *Spiroplasma* were most abundant in ant samples from Italy (i.e. where Wolbachia was not abundant). Note: red dashed line corresponds to the threshold of (10 copies in qPCR reaction; see Appendix S3, Table S3).
