# Supplementary material for: Bacterial communities within Phengaris (Maculinea) alcon caterpillars are shifted following transition from solitary living to social parasitism of Myrmica ant colonies
Source: Ecol Evol. 2019 Apr 2;9(8):4452–64. doi: 10.1002/ece3.5010 (PMC6476763; doi:10.1002/ece3.5010)
Supplement: Supplementary file 3 [file ECE3-9-4452-s003.docx]

# APPENDIX S3 – SUPPLEMENTARY TABLES

**Table S1** | **Samples collected for this study.** A total of 13 *P*. *alcon* caterpillars on plants and 9 caterpillars inside *Myrmica schencki* colonies were sequenced. Ant worker and ant larva extractions were pooled on a per-nest basis before sequencing.

|  | **Caterpillars on Plants** | **Caterpillars in ant nests** | **Ant workers** | **Ant**  **larvae** | ***Gentiana* buds** | **Soil** |
| --- | --- | --- | --- | --- | --- | --- |
| ES | 4 | 0 | 0 | 0 | 0 | 0 |
| CH | 4 | 4 | 4; pooled | 4; pooled | 2 | 1 |
| IT | 5 | 5 | 8; pooled  (2 colonies) | 10; pooled  (2 colonies) | 2 | 2 |
| n = | **13** | **9** | **3** | **3** | **4** | **3** |

**Table S2** | Mean abundances of OTUs with significant differences in abundance among caterpillar and ant groups (G-test on CSS-normalized abundances; Bonferroni *p*< 0.05). CSS-normalized quantities are given below; unlike raw or rarefied read counts, these normalized data account for under-sampling, without discarding reads. **The most differentially abundant species were two potentially symbiotic *Spiroplasma*, but two putatively environmental bacteria (a *Raoultella* sp. and *Rahnella woolbedingensis*) were also significantly differentially represented between groups.**

| **Taxonomy** | **Bonferroni**  ***p*** | ***P. alcon* (plant) CH** | ***P. alcon* (plant) IT** | ***P. alcon* (ant colony) CH** | ***P. alcon* (ant colony) IT** | ***M. schencki* worker CH** | ***M. schencki* worker IT** | ***M. schencki* larvae CH** | ***M. schencki* larvae IT** |
| --- | --- | --- | --- | --- | --- | --- | --- | --- | --- |
| *Spiroplasma* sp. [2] | 5.90E-05 | 0 | 1.278562 | 0 | 0.789844 | 0 | 9.86535 | 0 | 10.80535 |
| *Spiroplasma* sp. [1c] | 0.001356 | 1.828575 | 4.60256 | 3.5892 | 2.69476 | 0.76881 | 16.3465 | 2.1472 | 15.297 |
| *Raoultella* sp. | 0.008408 | 0 | 0 | 0 | 0.193046 | 0 | 0 | 9.9423 | 0 |
| *Rahnella woolbedingensis* | 0.03981 | 0.9883 | 1.085114 | 1.6614 | 2.257254 | 0.25334 | 0.828145 | 13.777 | 0.682115 |

**Table S3** | **Primers and standard curve characteristics for qPCR experiment.**

| **Target**  (gene name and accession no.) | **Sequence (5’ to 3’)** | **Tm (°C)^a^** | **Amplicon size** | **Standard curve** | | |
| --- | --- | --- | --- | --- | --- | --- |
|  |  |  |  | **Efficiency,**  **R^2^** | **Slope, intercept** | **LOD Cq^b^**  **#copies** |
| **Bacteria**  **(*16S rRNA gene*)** | **F: AGGATTAGATACCCTRGTAGTC**  **R: CATSMTCCACCRCTTGTGC** | **58.4 - 60.1**  **57.5 - 61.6** | **varies** | **1.858 =**  **85.8 %, 0.997** | **-3.716,**  **38.231** | **31.386**  **100** |
| ***P. alcon EF1a* (HQ918094.1)** | **F: CCCCAGCTAACATCACTACTG**  **R: AACGTATCCACGACGCAACTC** | **61.2**  **61.2** | **134 bp** | **1.905 =**  **90.5 %, 0.999** | **-** | **-** |
| ***Wolbachia* in *P. alcon***  **(*16S rRNA gene*,**  **this study)** | **F: AAGGCGTCTATCTGGTTCAAATC**  **R:TCCCATATTTAACATTCATCGTTTAC** | **60.9**  **60.1** | **110 bp** | **1.96 =**  **96 %, 1.000** | **-3.420,**  **36.446** | **32.988**  **10** |
| ***Spiroplasma* from *Myrmica* workers**  ***(16S rRNA gene*,**  **this study)** | **F: TCGAACGGGGTGCTTGCAC**  **R: CTAATACGCCGCATCCTCATC** | **61.6**  **61.2** | **176 bp** | **1.905 =**  **90.5 %, 0.999** | **-3.574,**  **37.939** | **34.523**  **10** |

^a^ Melting temperatures were calculated with the online tool described in Kibbe (2007).

^b^ *Cq, quantification cycle,* previously known as the threshold of detection (Ct) (Bustin et al. 2009)*. LOD* refers to the limit of detection of a primer set, here expressed as the Cq value at which the lowest number of plasmid copies were detected.
